# Effect of Pheromones, Plant Volatiles and Spinosad on Mating, Male Attraction and Burrowing of *Cadra cautella* (Walk.) (Lepidoptera: Pyralidae)

**DOI:** 10.3390/insects11120845

**Published:** 2020-11-28

**Authors:** Abeysinghe M. P. Sammani, Dissanayaka M. S. K. Dissanayaka, Leanage K. W. Wijayaratne, Thushara C. Bamunuarachchige, William R. Morrison

**Affiliations:** 1 Department of Plant Sciences, Faculty of Agriculture, Rajarata University of Sri Lanka, Puliyankulama, Anuradhapura 50000, Sri Lanka; prabodha12sammani@gmail.com (A.M.P.S.); dissanayaka.randeniya@gmail.com (D.M.S.K.D.); 2 Department of Bioprocess Technology, Faculty of Technology, Rajarata University of Sri Lanka, Mihintale, Anuradhapura 50000, Sri Lanka; heroiraj@gmail.com; 3 USDA Agricultural Research Service, Center for Grain and Animal Health Research, 1515 College Ave., Manhattan, KS 66052, USA; william.morrison@usda.gov

**Keywords:** *Cadra cautella* mating disruption, sex pheromone, botanicals, burrowing, spinosad

## Abstract

**Simple Summary:**

Sex pheromones used at higher concentrations than their availability in insects have been successful in decreasing the insect population. Furthermore, botanical oils have also had negative impacts on the insect population level. In the first study, we investigated the combined effect of sex pheromones and plant oils on mating of almond moth. The second study sought the burrowing ability of almond moth larvae through different flour media. Mating declined on the presence of pheromones and botanical oils, whereas burrowing ability differed with flour type. Exposure to spinosad affected both mating success and larval penetration through flour media. The findings of this study reveal that various applications of sex pheromones, plant oils and spinosad ensure the better protection of cereal-based agricultural produce from insect infestation during storage.

**Abstract:**

Mating disruption of *Cadra cautella* (Walk.) (Lepidoptera: Pyralidae) using its sex pheromone components, (Z, E)-9,12-tetradecadienyl acetate (ZETA) and (Z)-9-tetradecadien-1-yl acetate (ZTA), is successful in its population management. In addition, botanical oils have extensively been investigated in stored product pest management, but the effect of synthetic sex pheromones on the mating of *C. cautella* in the presence of plant volatiles is still unknown. Spinosad is used in food facilities as a contact insecticide but, if *C. cautella* larvae burrow into food, they may escape from spinosad. Importantly, the impact of spinosad on burrowing ability of *C. cautella* remains unknown. Therefore, the objectives of this study were to determine the effects of sex pheromone components ZETA and ZTA in the presence of botanical oils on the mating of *C. cautella* and the burrowing ability of *C. cautella* larvae in different types of flour treated with spinosad. In the first study, male and female moths were introduced into the cubicle having botanical oils and pheromone components. The mating status of female moths and male moth attraction to the trap was determined. The control experiments had only the botanical oils or pheromones. In the second study, burrowing ability of *C. cautella* larvae through different flour types was evaluated over 10 d. The flour was sprayed with spinosad (treatments) or water (controls). The mating success was higher with botanical oils alone but declined with exposure to pheromone either alone or combined with botanical oils. No differences in male attraction to traps were observed in botanical only, pheromone only or pheromone + botanical oil treatments. The burrowing of *C. cautella* larvae differed with flour type and spinosad altered burrowing ability. Thus, we conclude that the mating and burrowing of *C. cautella* is influenced by its pheromone and by exposure to botanicals and spinosad.

## 1. Introduction

There is an increasing demand for food due to the rapid increase of world population by 83 million people every year [1]. Post-harvest protection of cultivated food crops is an essential component to cater this food requirement. Indeed, post-harvest losses of agricultural commodities are very high, as up to 1/3 of the commodities produced are lost annually across the world, which amounts to 1.3 billion tons of food [2]. Insects are a major cause of deterioration of stored food, which may even be higher in tropical locations [3]. In Sri Lanka, serious losses due to insect infestation are reported for stored grains [4,5,6], rice flour [7], spices [8] and animal feed [9].

*Cadra cautella* (Walk.) (Lepidoptera: Pyralidae) is an important pest for storage losses in Sri Lanka [10] and other countries [11,12,13]. It damages grains and grain-based products, pulses, dried fruits, nuts, spices, cocoa beans and confectionaries [11,13,14,15,16,17,18]. The common control methods used for *C. cautella* and other stored-product insects are typically contact insecticides [19], fumigants [20,21,22], high temperature [23], and aeration practices [24]. Despite the success of these methods, each has its own drawbacks, which may variously include insecticide toxicity, health hazards to workers [25], non-target effects on the environment [26,27] and the development of resistance in insects [28]. Therefore, there has been a push to develop biorational pest management methods in the postharvest supply chain [27,29,30,31].

Monitoring and suppressing insect populations are important parts of integrated pest management program (IPM) [32,33,34]. Most often, pheromone traps and mating disruption are used to monitor and control the population of *C. cautella* [35,36,37]. Female moths of *C. cautella* release the two-component sex pheromone, (Z, E)-9,12-tetradecadienyl acetate (ZETA) and (Z)-9-tetradecadien-1-yl acetate (ZTA) in a natural 14:1 ratio [38]. ZETA is the major component and ZTA is a synergist of ZETA [39]. The synthetic form of ZETA is commercially available and used in traps for monitoring *C. cautella* [15,40,41]. Recent studies on different uses of *C. cautella* pheromones and its implications for behavioral manipulation [42] have augmented the information available to food facility managers in Sri Lanka to support the use of this technology in IPM programs.

Botanical compounds have extensively been investigated for application in stored-product insect pest management. Many studies have documented the ability of botanical insecticides to induce mortality, repellency, and other behavioral changes in stored-product insects [43,44,45,46,47,48,49,50]. In fact, some of the prior work has specifically evaluated the direct effects of botanical compounds on *C. cautella* [51,52]. However, despite the work evaluating the effect of botanicals on *C. cautella* and the extensive amount of research conducted on different uses of pheromone components of *C. cautella* [15,42], there have been few studies elucidating the potentially synergistic effects of using pheromones and plant volatiles (e.g., with botanical oils as a surrogate) on *C. cautella* mating disruption. However, it is reported that the rise of systems-level thinking in agriculture has resulted in significant innovation in food production [53]. Thus, it is increasingly important not just to evaluate individual tactics independently of each other, but in combination, to deliver a fuller understanding of postharvest pest management systems.

In *C. cautella*, larvae feed on durable goods and contribute to contamination, decreasing the quality of commodities to unacceptable levels for human consumption [16]. Though anecdotally it is known that *C. cautella* frequently burrow, there has been little formal study of this behavior in food sources [54]. Lack of knowledge on the burrowing ability of *C. cautella* may lead to inaccurate predictions of population development after exposure to contact insecticides, as larvae may be able to escape exposure in refugia found beneath the surface of food sources.

One potential contact insecticide that may affect burrowing of *C. cautella* is spinosad. Spinosad is a biorational, reduced-risk insecticide developed from the bacterium *Saccharopolyspora spinosa* [55]. It is effective at controlling mature and immature stages of various stored-product insect species [3,56,57,58,59]. Spinosad has also shown promise in controlling *C. cautella* [57]. However, the effect of exposure of *C. cautella* larvae to spinosad and its effect on burrowing ability has not been investigated. Therefore, the objectives of this study were to determine the effect of sex pheromone components with or without botanical oils on the mating of *C. cautella* moths, and to determine the burrowing ability of *C. cautella* larvae in different types of flour treated with spinosad or water.

## 2. Materials and Methods

### 2.1. Insect Cultures

The laboratory culture of *C. cautella* was established using adult moths collected from a rice milling center at Puliyankulama, Anuradhapura, Sri Lanka. The adult insects were introduced to rice flour medium in a 5 L plastic bottle with a perforated lid (50 adults on 250 g of rice flour). These *C. cautella* cultures were kept inside an incubator with complete darkness (FH-1200 LED T8, HiPoint Laboratory, Taiwan) at a constant 33 ± 1 °C temperature and a 60 ± 5% relative humidity.

### 2.2. Experiment 1. Response of Cadra cautella to Sex Pheromone Components (Z, E)-9, 12-tetradecadienyl Acetate (ZETA) and (Z)-9-tetradecadien-1-yl Acetate (ZTA) in the Presence of Botanicals

#### 2.2.1. Preparation of Adults

*Cadra cautella* adults were sexed during the pupal stage by referring to the two nodes present on the ventral side of the 8th segment of male pupa under a dissecting microscope (OPTIKA, Triace, Italy) [60]. Individual male and female pupae were placed in separate plastic bottles (3.6 cm diameter and 6.2 cm height) containing 5 g of rice flour. These adults emerged in the bottles and were used in the experiments.

#### 2.2.2. Cubicle Construction

Four cubicles (1.5 m × 1.5 m × 1.5 m length:width:height) were constructed using metal frames. The top, bottom and two opposite sides of each cubicle were covered by transparent polythene (25 mm thickness). The remaining two opposite sides of each cubicle were covered using an insect proof net (mesh size: 300 holes/cm^2^) to allow exchange of air from inside the cubicle and the outside environment. Velcro (Garment Accessories.lk, Rajagiriya, Sri Lanka) was used to attach the polythene and net to the cubicle.

#### 2.2.3. Preparation of Pheromone Blend

The pheromone blend of ZETA: ZTA used in this study was 5:1, which demonstrated the highest mating disruption in prior work [42]. The pheromone blend was prepared by diluting a commercially available neat stock solutions of ZETA (100%) (100 μL) and ZTA (100%) (20 μL) (Insects Ltd. Inc., Westfield, NJ, USA) in hexane (100 μL) using a micropipette (Labnet International Inc., Poland) [60]. Four replicate pheromone solutions were prepared. The loading rate of 90 mg of ZETA per dispenser approached the commercial standard loading rate of 160 mg in Cidetrak™ mating disruption dispensers (Trece, Inc., Adair, OK, USA) [61], and reflected concentrations used in analogous work in the recent past for continuity [42].

#### 2.2.4. Botanical Oils

The following botanical oils were used: camphor oil (*Cinnamomum camphora*), sandalwood oil (*Santalum album*), neem oil (*Azadirachta indica*), cinnamon oil (*Cinnamomum verum*), citronella oil (*Cymbopogon nardus*) and mee oil (*Madhuca longifolia*) (LBK Aushadalaya, Anuradhapura, Sri Lanka). Neat 2 mL of each botanical oil was measured by using a micropipette (Labnet International Inc., Edison, NJ, USA) and added into separate plastic bottles (3.6 cm diameter and 6.2 cm height).

#### 2.2.5. Introduction of Botanical Oils and Pheromone Blend into the Cubicle

The plastic bottle containing the botanical oil was placed at the middle of the bottom side of the cubicle. In the control experiment 1, the cubicle contained the botanical oil alone (no pheromone components). In the control experiment 2, the cubicle contained only the pheromone blend (without botanical oils). A mixture consisting a total of 120 μL of ZETA and ZTA pheromone components (in a 5:1 ratio as described above) was added to a triangular proprietary, slow-release filter paper (8 cm^2^) (Trece, Inc., Adair, OK, USA) and attached to a piece of rigiform using a needle, which was then placed on a Petri dish (80 × 15 mm). The Petri dish was located inside a monitoring trap (Storgard^®^ kit insect monitoring system, Trece Inc., Adair, OK, USA), which was hung from the center suspended 75 cm distance from the top [62]. Finally, the treatment trials contained both botanical oils and the pheromone blend placed in the same manner as described above in the control experiments. Three hours following the placement of the botanicals, pheromone blend, or both inside the cubicle, the moths were introduced. The four replicates of a particular experiment were conducted using the four cubicles at the same time. The experiments using four replicates of botanical only (six botanicals); pheromone only and botanical + pheromone treatment categories were conducted separately.

#### 2.2.6. Introduction of *Cadra cautella* Moths to the Cubicle

The experiment was conducted using 4 cubicles in a completely randomized design (CRD). A population size of 20 moths (10 males, 10 females), which was found to be most susceptible to mating disruption from prior work [42], was used in this experiment. Moths were 2–4 d old, had emerged individually in bottles from the larvae introduced, when they were individually introduced into the cubicles. After 24 h, the moths in each cubicle were collected separately using an aspirator into conical flasks (250 mL). The moths were frozen at −10 °C for 1 h [62] to ensure the death of insects as well as the best visibility of spermatophores [63]. The mating status of moths was determined by observing the presence of spermatophores in the bursa copulatrix of female moths [64]. To accomplish this, moths were dissected under a stereomicroscope (OPTIKA, Triace, Italy). The number of female moths that were mated following each exposure was recorded. Furthermore, the number of male moths captured inside monitoring traps was determined.

Following each experiment, the entire facility and the cubicles were washed using a biodegradable detergent (Britol Disinfectant Pine, Antler Industries Pvt. Ltd., Piliyandala, Sri Lanka) to remove any pheromone residues and was allowed to air dry before the next round of experiments. Furthermore, before the next experiment, the contaminated air inside the room was removed using an exhaust fan.

#### 2.2.7. Recording Environmental Conditions of Cubicles

The temperature and relative humidity profiles inside and outside the cubicles during the experiment were recorded by data loggers (TM-305U, Tenmars Electronics Co., Ltd., Taiwan). The average values of temperature and relative humidity inside and outside of the cubicles were 30 ± 1 °C, 63 ± 1% RH and 32 ± 1 °C, 64 ± 2% RH, respectively.

#### 2.2.8. Experimental Design and Data Analysis

The experiment was conducted as a completely randomized design (CRD). Each control or treatment experiment had four replicates. Responses consisted of females (mated or unmated) and males (captured or not), which were analyzed using a generalized linear model based on a binomial distribution with a logit link function. Explanatory variables included the presence of pheromone (yes or no), botanical oils (as above), and their interaction. Overdispersion was checked and not found to be a problem. Upon a significant result from the model, χ^2^-tests with Bonferroni correction to the *p*-value were used for multiple comparisons.

### 2.3. Experiment 2. Burrowing of Cadra cautella Larvae in Different Flour Media Treated with Spinosad

#### 2.3.1. Insects

*Cadra cautella* larvae aged 10 d were used in this experiment. These larvae were obtained from *C. cautella* cultures reared as described in Section 2.1. For this experiment, larvae measuring approximately the same size were used.

#### 2.3.2. Types of Flour and Insecticide Application

The following types of flour were purchased from a local retailer and used in the experiment: rice flour, maize flour, mung bean flour, cowpea flour and atta flour (hard wheat flour). For each replicate of the types of flour, 250 g of that specific flour was weighed into separate plastic sealable bags (20 × 30 cm width:length). The commercial product ‘Success’ (Spinosad 25 g/L SC, Hayleys Agriculture, Sri Lanka) was used as the source of spinosad. A solution with the labeled rate of 25 ppm of spinosad was prepared by diluting the 100 µL of the commercial product in 100 mL distilled water. Next, each replicate 250 g of flour weighed above was placed on a piece of aluminium foil (approx. 1 mm thickness). A total of 3.75 mL of the spinosad solution was sprayed on to each 250 g flour sample by using an artist’s air brush (VL-202S, Paasche Airbrush Company, USA) [65]. Immediately afterwards, each flour sample was placed inside a sealable bag and hand tumbled for 1 min. In the control treatment, each 250 g flour sample was instead sprayed with 3.75 mL of distilled water in the same way. There were four replicate flour samples from each flour type and treatment combination. For each replicate of flour type and treatment, 40 g of treated material was used in to determine the burrowing depth of *C. cautella* larvae, as described below.

#### 2.3.3. Introduction of *Cadra cautella* Larvae to Flour

Glass vials (50mL capacity and measuring 3 cm diameter, 20 cm height) were used to assess the burrowing behavior for the experiment. The volume level filled by 40 g of flour without compaction was marked on the circumference of the vial (approx. at 18 cm from bottom). Four vertical lines in each cardinal direction, including North (A), South (B), East (C) and West (D), were marked on the circle. These points served as reference points for noting the burrowing of *C. cautella* larvae through a given type of flour. The treatments consisted of spinosad-treated (different types as mentioned in 2.3.2) or water-treated flour. Twenty *C. cautella* larvae (10 d old) were introduced into each vial. The vials were maintained inside an incubator (FH-1200 LED T8, HiPoint Laboratory, Taiwan) at 33 ± 1 °C and 60 ± 5% RH. In each vial, the daily average burrowing depth of *C. cautella* larvae was determined by measuring the distance burrowed from the reference points (A, B, C and D) along the vertical lines. In addition, the total cumulative burrowing depth was noted as the total cumulative distance burrowed over 10 d.

#### 2.3.4. Experimental Design and Data Analysis

The experiment was conducted as a completely randomized design (CRD). Each treatment or control experiment had four replicates. Differences in the cumulative burrowing depths of *C. cautella* larvae in different types of flour were analyzed by using ANOVA procedures of statistical analysis system (SAS) [66] with mean separation by Tukey’s test and significance at α = 0.05.

## 3. Results

### 3.1. Experiment 1. Response of Cadra cautella to Sex Pheromone Components (Z, E)-9, 12-tetradecadienyl Acetate and (Z)-9-tetradecadien-1-yl Acetate in the Presence of Botanicals

**Figure 1:**
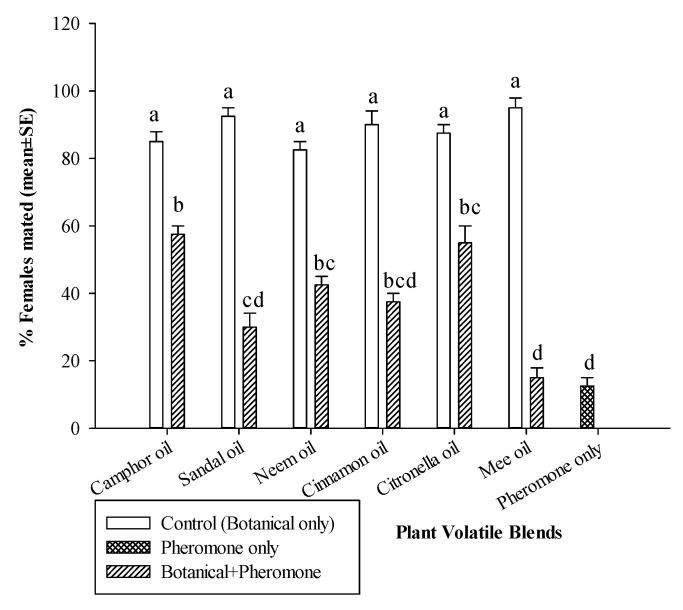
Percentage (mean ± SE) of mated *C. cautella* females following exposure to plant volatiles (e.g., botanical oils) only (white bars), pheromone only (hatched bars), or both together (diagonal lined bars) (*n* = 4). Means followed by the same letter are not significantly different (Tukey’s test following ANOVA, α = 0.05).

**Figure 2:**
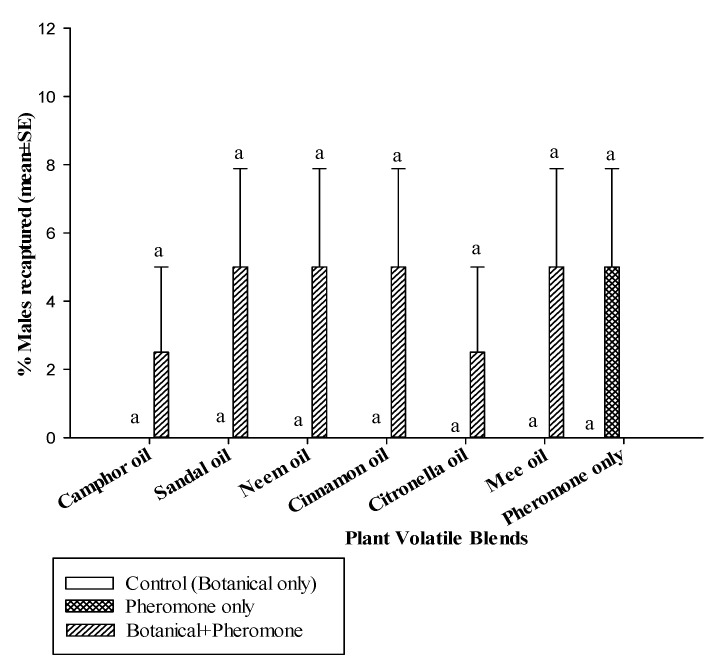
Percentage (mean ± SE) of *C. cautella* males attracted to plant volatiles (e.g., botanicals) only (white bars), pheromone only (hatched bars), or both together (diagonal lined bars) expressed as a percentage of those released that were recaptured (*n* = 4 per bar). All the treatments using botanical oil only had no males captured (0% of total released). Means followed by the same letter are not significantly different (Tukey’s test following ANOVA, α = 0.05).

The presence of pheromone (GLM: *χ*^2^ = 38.0, df = 1, *p* < 0.0001), but not the botanical oils alone (*χ*^2^ = 2.14, df = 6, *p* = 0.14), significantly affected the number of mated females (Figure 1). In general, the percentage of mated females was significantly higher in the botanical oil only controls than the botanical oil + pheromone treatment (interaction: *χ*^2^ = 15.8, df = 6, *p* < 0.01) (Figure 1). There were no significant differences in the percentage of mated females among the different botanical oils when tested alone. When the botanical oils were combined with pheromone, the percentage of mated females was higher in the presence of camphor oil (57.5 ± 2.5%), citronella oil (55 ± 5.0%) and neem oil (42.5 ± 2.5%) compared with sandalwood oil (30 ± 4.08%) and mee oil (15 ± 2.89%). The percentage of mated females in cinnamon oil (37.5 ± 2.5%) was in-between these two categories. Mating success was not different among sandalwood oil, mee oil and cinnamon oil when compared to the control treatment (12.5 ± 2.5%), which used the pheromone blend only. Neither the presence of pheromone (*χ*^2^ = 1.39, df = 1, *p* = 0.24), the botanical oils (*χ*^2^ = 7.78, df = 6, *p* = 0.25), nor their interaction (*χ*^2^ = 10.8, df = 6, *p* = 0.09) affected male attraction (Figure 2).

### 3.2. Experiment 2. Burrowing of Cadra cautella Larvae in Different Types of Flours Treated with Spinosad

In all the flour types, the burrowing of *C. cautella* larvae gradually increased as more time elapsed. In the water-treated controls, the burrowing depth varied from 0.36 ± 0.02 cm (atta flour) to 2.04 ± 0.15 cm (maize flour). In the spinosad-treated flour, the minimum and maximum burrowing depths were 0.69 ± 0.02 cm (atta flour) and 3.2 ± 0.14 cm (rice flour), respectively. Overall, the burrowing of *C. cautella* larvae was higher in the spinosad-treated flour than their water-treated controls, except for maize flour (F_9,30_ = 85.2, *p* < 0.0001) (Table S1). In maize flour, *C. cautella* burrowed a 1.3–1.6-fold greater depth in the water-treated control than in the spinosad-treated flour, regardless of the duration.

In rice flour, mung bean flour and cowpea flour, there was a significant difference in the burrowing depth between the spinosad-treated and water-treated flour (Table S1). Specifically, larvae burrowed 2.3–4.1-fold, 2.1–2.0-fold, and 1.9–2.2-fold more in spinosad-treated compared to water-treated rice, mung bean, and cowpea flour, respectively. In maize flour and atta flour, there were no significant differences observed between the spinosad-treated and control.

## 4. Discussion

The mate-finding behavior of lepidopterans mainly depends on males following sex pheromone plumes to their point source (e.g., females) [67,68]. Nansen and Phillips [69] found that many of the botanical oils enhanced the oviposition of *Plodia interpunctella* (Hübner) (Lepidoptera: Pyralidae), the Indianmeal moth, including olive, sunflower and corn oils. Previous work has found that plant volatiles stimulate the release of the female ermine moth’s sex pheromones to attract males (e.g., Yponomeuta) [70]. In this study, we found that a greater percentage of *C. cautella* successfully mated in the presence of camphor oil, citronella oil and neem oil, which supports prior findings that plant volatiles may modulate mating success among moths. These studies have shown that plant volatiles may synergize attraction to their natural sex pheromones [69,70,71,72,73]. In this case, decreased mating disruption efficacy may be because of an effect by botanical oils on (1) males, (2) females, or (3) both sexes. In future work, the specific target sex behavior and mechanism should be investigated in order to deliver a better understanding of how these biorational approaches may interact with each other. While we used a 5:1 ratio of ZETA:ZTA in this study, the natural ratio produced for these by *C. cautella* is 14:1; addition of botanical oils could have changed the effective concentration of the lure in the cubicles, with ramifications for mate-finding. In other systems, there may be reduced responses to pheromone after exposure to plant or non-host volatiles some non-Lepidopteran taxa, as was shown to be the case with *Dendroctonus frontalis* LeConte (Coleoptera: Scolytidae), the southern pine beetle, following exposure to 4-allyl anisole in Loblolly pine (*Pinus taeda*), and for some other bark beetle species [74,75,76]. Nonetheless, in the laboratory setting evaluated here, the use of botanical oils for control of stored product lepidoptera may inadvertently circumvent other pest management tactics, such as mating disruption. Future research should explore this behavioral phenomenon at a semi-field and larger scale, as well as explore the mechanisms that enhance the mating in *C. cautella* following exposure to plant volatiles, considering multiple tactics together may have important implications for their implementation for food facilities.

The chemical (Z,E)-9,12-tetradecadienyl acetate secreted by *C. cautella* is also emitted by the unrelated taxon *Spodoptera exigua* (Hübner) (Lepidoptera: Noctuidae), the beet armyworm. The plant volatiles linalool and myrcenebenzaldehyde have been shown to increase the response of male *S. exigua* to their natural sex pheromone [75]. However, we found no evidence to support increased male attraction in the presence of plant volatiles, at least for the specific botanical oils tested. It is possible that individual components in the headspace blends from the botanical oils may be behaviorally antagonistic, which results in a net effect of no response, as has been shown for other species such as the invasive spotted-wing drosophila, *Drosophila suzukii* (Matsumura) (Diptera: Drosophilidae) [77]. It may be useful to test the behavioral effects of individual volatiles instead of blends produced by the botanical oils, or at least screen the volatiles in the blends produced by the botanical oils with electroantennography coupled with gas chromatography.

The current study revealed that *C. cautella* readily burrowed in different types of flour. Prior to this work, little information was available on the burrowing ability of *C. cautella* larvae through different commodities. Bell [78] and Mullen et al. [79] showed that mature *C. cautella* larvae will wander in the food and may bore through packaging material. It has also been documented that *C. cautella* larvae can penetrate through a variety of packing materials, such as cellophane, polyethylene, paper material, polyvinylchloride and polypropylene films [80,81,82]. *P. interpunctella*, a closely related lepidopteran species that shares certain behavioural characteristics with *C. cautella* [16] is also able to penetrate through polyethylene [83,84], but this ability differs with the age of larvae [81,82,85] and the thickness of material [82,85]. By contrast, larvae of *P. interpunctella* and *Trogoderma variabile* Ballion (Coleoptera: Dermestidae) cannot penetrate through methoprene-treated foil packaging [84]. Some other studies have revealed that the few warehouse beetle *T. variabile* larvae that were able to penetrate methoprene-treated foil experienced deformities in the adult stage [86]. The use of packaging as a cultural control tactic for *C. cautella* may be worth considering in combination with behaviorally based tactics, such as mating disruption and other biorational strategies in future research.

Our study has shown that spinosad-treated flour results in elevated burrowing activity for *C. cautella* larvae compared with water-treated controls. This suggests that burrowing behavior by *C. cautella* is flexible and inducible, and may result in behavioral resistance to the use of contact insecticides, whereby larvae will escape by seeking out refugia deeper in commodities. This may severely curtail the actual efficacy of contact or residual insecticides when used to treat sites of spillage or bulk commodities with existing infestations of *C. cautella*. Furthermore, unless commodities are thoroughly examined during cleaning and remedial actions, *C. cautella* infestation may continue unabated until the population increases to higher levels and flying adults emerge. Furthermore, burrowing might negatively affect the pheromone and kairomone-baited trapping of conspecific *C. cautella* when contact insecticides are used in a food facility, because their application will push individuals further into commodities. However, adult moths will likely not be affected, so monitoring in long-standing infestations or for flight activity traps may not be impacted. Future experiments need to investigate the interaction between contact insecticides and monitoring for *C. cautella*.

The current study found how the burrowing behavior of *C. cautella* larvae is affected by spinosad. It was also found that once adults emerged, their mating can be disrupted by exposure to pheromone and botanical oils used either alone or in combination. Previous work has shown how *C. cautella* larvae can be controlled by spinosad [42]. Even if *C. cautella* larvae seek refuge in flour treated with spinosad, and thus escape monitoring, their population can still be managed once they emerge as adults by using pheromones, botanicals or their combination as highlighted in the current work. Overall, this work illuminates the behavior and mating of *C. cautella*, and particularly how it is influenced by biorational pest management strategies. This information may be used for protecting stored food from infestation by this devastating pest. 

## 5. Conclusions

In conclusion, camphor oil, citronella oil and neem oil increase the mating success of *C. cautella* in the presence of the synthetic sex pheromone. The burrowing depth of *C. cautella* larvae varies with the type of flour, but generally is increased in spinsoad-treated flour compared to controls for rice, mung bean, and cowpea flour.

## Supplementary Materials

The following are available online at https://www.mdpi.com/2075-4450/11/12/845/s1, Table S1: Cumulative burrowing depth (mean ± SE) by *Cadra cautella* larvae over 10 days through different flour media treated with spinosad (*n* = 4).
