# Supplementary material for: Effect of Pheromones, Plant Volatiles and Spinosad on Mating, Male Attraction and Burrowing of Cadra cautella (Walk.) (Lepidoptera: Pyralidae)
Source: Insects. 2020 Nov 28;11(12):845. doi: 10.3390/insects11120845 (PMC7760550; doi:10.3390/insects11120845)
Supplement: Supplementary file 1 [file insects-11-00845-s001.pdf]

Table S1. Cumulative burrowing depth (mean±SE) by *Cadra cautella* larvae over 10 days through different flour media treated with spinosad (n=4).

		**Cumulative depth (mean±SE) burrowed through different types of flour (cm)**									
		**Duration of burrowing (Days)a**									
Treatment	Flour medium	1	2	3	4	5	6	7	8	9	10
Spinosad treated	Rice	1.556±0.1a	2.4±0.10a	2.538±0.10a	2.638±0.0a	2.788±0.11a	2.844±0.10a	2.931±0.10a	3.138±0.15a	3.2±0.14a	3.2±0.14a
	Maize	0.663±0.0de	0.738±0.1d	0.806±0.10def	0.919±0.1cd	1.006±0.16cde	1.138±0.16de	1.325±0.18bc	1.438±0.17bc	1.525±0.15bc	1.631±0.16bc
	Mungbean	0.856±0.1cd	1.119±0.0bc	1.125±0.06bcd	1.156±0.0bc	1.181±0.06bcd	1.188±0.05cde	1.281±0.05c	1.325±0.04c	1.431±0.05c	1.481±0.05c
	Cowpea	1.225±0.0ab	1.344±0.0b	1.425±0.05b	1.463±0.0b	1.531±0.05b	1.563±0.05bc	1.625±0.04bc	1.638±0.04bc	1.656±0.04bc	1.675±0.04bc
	Atta	0.556±0.0def	0.6±0.02d	0.65±0.02f	0.669±0.0de	0.681±0.01ef	0.688±0.02fg	0.688±0.02de	0.688±0.02de	0.688±0.02de	0.688±0.02de
Control	Rice	0.375±0.03ef	0.888±0.02cd	1.019±0.01cde	1.169±0.02bc	1.306±0.03bc	1.388±0.03bcd	1.419±0.04bc	1.419±0.04bc	1.419±0.04c	1.419±0.04c
	Maize	1.063±0.12bc	1.194±0.11bc	1.269±0.13bc	1.406±0.13b	1.544±0.16b	1.638±0.17b	1.731±0.17b	1.831±0.15b	1.944±0.15b	2.044±0.15b
	Mungbean	0.425±0.05ef	0.563±0.06de	0.581±0.06fg	0.6±0.05de	0.606±0.06ef	0.65±0.04fg	0.656±0.04de	0.656±0.04de	0.706±0.03de	0.706±0.03de
	Cowpea	0.563±0.04def	0.631±0.05d	0.706±0.04ef	0.763±0.03d	0.8±0.04de	0.819±0.03ef	0.85±0.04d	0.856±0.03d	0.863±0.03d	0.863±0.03d
	Atta	0.213±0.01f	0.244±0.01e	0.294±0.01g	0.325±0.02e	0.356±0.02f	0.363±0.02g	0.363±0.02e	0.363±0.02e	0.363±0.02e	0.363±0.02e
	**P value**	**<0.0001**	**<0.0001**	**<0.0001**	**<0.0001**	**<0.0001**	**<0.0001**	**<0.0001**	**<0.0001**	**<0.0001**	**<0.0001**
	**F value**	**31.48**	**79.21**	**79.79**	**80.24**	**66.32**	**67.97**	**68.26**	**78.19**	**86.69**	**85.18**

[a]For a given duration of burrowing, means followed by the same letter are not significantly different at α=0.05 according to Tukey's test following ANOVA.
